# Electrochemical and spectral studies of auto-assembled arrays of calix[4]arenequinhydrone charge-transfer complex on indium–tin oxide (ITO) glass

**DOI:** 10.1007/s10847-013-0361-7

**Published:** 2013-09-13

**Authors:** Oumayma Ben Youchret-Zallez, Salma Besbes-Hentati, Marcel Bouvet, Hechmi Said

**Affiliations:** 1 Laboratoire de thermodynamique et d’électrochimie, Faculté des sciences de Bizerte, 7021 Zarzouna, Bizerte, Tunisia; 2 UMR 5260, CNRS, Institut de Chimie Moléculaire de l’Université de Bourgogne (ICMUB), Dijon, France

**Keywords:** Charge-transfer complex, Electrochemistry, Calix[4]arenequinhydrone, UV–Vis spectroscopy, ITO electrode

## Abstract

A sensing materiel based on calix[4]arene molecules is electrochemically deposited on ITO electrode coated. A brown film was electrodeposited at a potential E_imp_ = –1.00 V versus SCE in acetonitrile solvent, however in dichloromethane solvent, a bluish film auto-assembled on the ITO electrode coated at a potential E_imp_ = −0.65 V versus SCE. Both films are subsequently analyzed by cyclic voltammetry and UV–Vis spectroscopy. This investigation shows that in acetonitrile solvent, the charge-transfer complex, calix[4]arenequinhydrone was formed in electrolytic solution and it was not self-assembled on the ITO electrode. The related UV–Vis spectrum shows a single absorption band towards a wavelength about 350 nm. The optical behaviour of the blue film shows two absorption bands: the first one appears on the first absorption band of the acceptor at 305 nm and the second one in the visible range at 502 nm. The band situated in the visible range correspond to a well-defined charge-transfer band indicating the presence of the charge-transfer complex, the calix[4]arenequinhydrone.

## Introduction

**Scheme 1:**
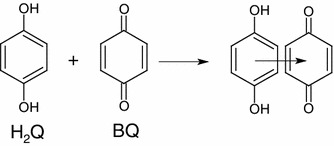
Schematic view of quinhydrone charge transfer from dihydroquinone and p-benzoquinone

Hydroquinone-quinone, donor–acceptor dyads (Scheme 1) are one of the most widely studied classes of compounds involving an electron transfer reaction [1]. The self-assembled supramolecular donor–acceptor systems have been constructed using either hydrogen bonding, ion pairing, cation complexation, or Van Der Waals interactions as binding mechanisms [2]. This result was backed up by researchers hwo reported that in quinhydrone, the charge-transfer interactions between the electron donor (hydroquinone), and the electron acceptor (quinone) stabilize the complex while additional stability may also be provided by hydrogen bonds [3–6]. The formation of complexes both in the solid and in solution from components which may reasonably be classified as electron donors and electron acceptors has long been recognized [7].

Various theories were developed at an early stage highlighted the formation of such complexes their presence was often recognized by their colours, and many theories concerning the forces stabilizing the ground state of the complex were confounded by explanations as to the nature of the transition which gives rise to the colour [8].

An electron donor–acceptor complex formed when a molecule of low ionization potential electron donor interacts with a molecule of high electron affinity (electron acceptor). Promotion of the electron from one of the upper filled orbital of the donor to an unfilled acceptor orbital gives rise to one or more electron absorption bands not observed to the donor or acceptor spectra. These usually formed charge-transfer bands and electron donor–acceptor complexes are classified according to the types of potentials involved in the charge-transfer interaction [8]. The molecules mentioned in this study produced from π–π electron donor–acceptor complexes are usually reversibly and rapidly formed and exhibit low energies of interaction [8].

The calix[4]arenequinhydrone studied in this work present a large similitude with the quinhydrone described earlier. Schierbaum et al. [9] and Adams et al. [10] have widely described the preparation and characterization of self-assembled calix[n]arenes but they have not implemented evidence the molecular orientation. Organic materials have been a great interest for their easy processing and interest sensing properties and their high luminescence efficiencies, which make them promising for many applications, such us chemical sensors [11, 12], thin film transistors [13, 14] and solar cells [15], and considered among the most important families of organic materials [16–18]. Several authors have shown that calix[n]arenes have good extractions properties due to their ability to recognize ions and organic molecules according to a key-lock interaction mechanism [19, 20].

From the electrochemical point of view, the adsorption of organic molecules at electrode–electrolyte interfaces can be considered as one of the most promising approaches not only for the preparation of ordered adlayers but also for elucidating the role of properties of adsorbed molecules [21, 22]. The electrochemical modification of ITO electrode surfaces with organic molecular films attracted a growing interest in various fields during the last decades. Domain of chemical sensors [23, 24] presents a potential application by operator the macrocyclic compounds as organic molecular materials.

In our preceding work, we show that the electrochemical reduction of di(methoxy-*p*-*tert*-butyl)calix[4]arenediquinone led to the corresponding calix[4]arenedihydroquinone [25]. The presence of both in the solution refers to the generation of calix[4]arenequinhydrone charge-transfer complex at the electrode surface through a donor/acceptor process type. Self-assembled adlayers of calix[4]arenequinhydrone was obtained by partial electrochemical reduction on the platinum electrode [25]. The calix[4]arenequinhydrone was obtained too in acetonitrile solution, by partial electrochemical oxidation of the calix[4]arenedihydroquinone [26].

The calix[4]arenequinhydrone charge-transfer complex was synthesized by chemical partial reduction of di(methoxy-*p*-*tert*-butyl)calix[4]arenediquinone by 0.5 equivalent of a reducing agent [27]. The calix[4]arenequinhydrone charge-transfer complex exhibits an intense solvatochromic absorption band in the visible region [27]. The charge-transfer complexes have been studied in solvents of low dielectric constant as described by Peover [28, 29] and its band energy has been characterized by UV–Vis spectroscopy [4].

**Scheme 2:**
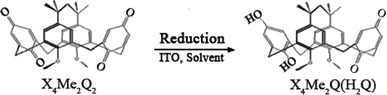
Schematic pathway of the electrochemical generation of X_4_Me_2_Q(H_2_Q) by the cathodic reduction of X_4_Me_2_Q_2_

The aim of this study is to find the accurate conditions to deposit electrochemically the calix[4]arenequinhydrone complex X_4_Me_2_Q(H_2_Q) adlayer on ITO electrode. This material is a charge-transfer complex formed by a partial reduction of the starting product, the calix[4]arenediquinone X_4_Me_2_Q_2_ (Scheme 2). The electrodeposition is carried out by varying the nature of the solvent. We use acetonitrile and dichloromethane as polar solvents having low dielectric constant and low dipolar moment. Before preparative electrolysis on ITO electrode, a preliminary study of the electroactivity domain is performed. This study allows us to locate the barriers of reduction and oxidation of the supporting electrolyte, to avoid the deterioration of the conductive layer ITO working electrode. Thus, the self-assembled adlayer on ITO glasses will be subsequently analyzed essentially by cyclic voltammetry and UV–Vis spectroscopy. We show that the color of the electrodeposited substance differs from brown to blue and depends on the nature and polarity of the solvent.

## Experimental part

Using the synthesis procedure developed by Van Loon et al. [30], we synthesized the dimethoxycalix[4]arene (X_4_Me_2_H_2_) from the calix[4]arene X_4_H_4_. Using the last form we synthesized the 5,17-di-*tert*-butyl-26,28-dimethoxycalix[4]arene-27,27-diquinone (X_4_Me_2_Q_2_) as described by Beer et al. [31].

Our target molecules are 5,17-di-*tert*-butyl-26,28-dimethoxycalix[4]arene-25,27-diquinone (X_4_Me_2_Q_2_), 5,17-di-*tert*-butyl-26,28-dimethoxycalix[4]arene-25,27-dihydroquinone (X_4_Me_2_(H_2_Q)_2_). X_4_Me_2_Q_2_ was considered as the starting product for the present electrochemical study.

All chemical manipulations were carried out under argon using Schlenk tube techniques. Acetonitrile and dichloromethane were distilled from CaH_2_ under argon. Diethyl ether was distilled from over sodium-benzophenone under argon. Trifluoroacetic acid and thallium (III) trifluoroacetate were purchase from strem chemicals and Alfa-Aesar, respectively, and used without further purification.

### Spectroscopic analysis

MALDI-TOF mass analysis was performed on a PerSeptive Biosystems (Framingham, MA, USA) Voyager Elite TOF mass spectrometer equipped with a nitrogen laser (λ = 337 nm). It was operated at 20 kV in the reflectron delayed extraction mode. The spectra were recorded in the absence of matrix. ^1^H NMR spectra were recorded on a Brucker AC-300 spectrometer. SEM technique was released by JEOL-JSM-5400 scanning microscopy.

### Synthesis of 5,17-di-tert-butyl-26,28-dimethoxycalix[4]arene-25,27-diquinone (X_4_Me_2_Q_2_)

**Fig. 1:**
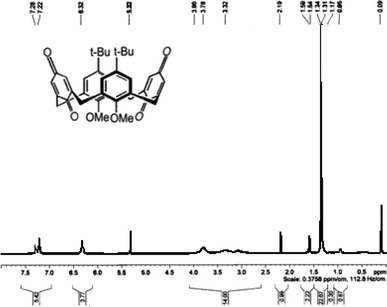
^1^H NMR spectrum unregistered for *p*-*tert*-butylcalixarene X_4_Me_2_Q_2_ in CDCl_3_

**Fig. 2:**
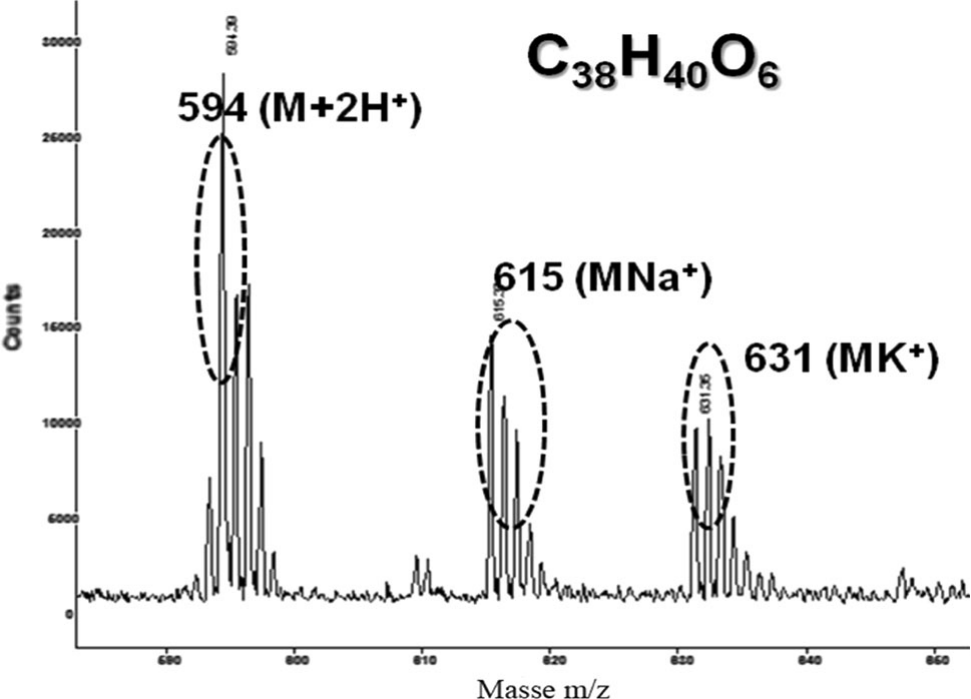
Mass spectrum of *p*-*tert*-butylcalixarene X_4_Me_2_Q_2_

The Synthesis of dimethoxycalix[4]arene X_4_Me_2_H_2_ was achieved according to the literature [30]. Several chromatographic separations were performed on silica gel 60 (SiO_2_, Merk, particle size 40–63 μm) and detected by an R410 refractometer. The detector used was an R410 refractometer. For the synthesis of X_4_Me_2_Q_2_, the P.D. Beer protocol was followed [31]. To a solution of Tl(OCOCF_3_)_3_ (2.42 g; 4.44 mmol) in trifluoroacetic acid (5 mL), 0.5 g (0.74 mmol) of (X_4_Me_2_H_2_) was added and stirred for 2 h in the dark and under argon. The trifluoroacetic acid was then removed in vacuum and the residue poured into ice-water (15 mL). The product was extracted with chloroform. The organic adlayer was then washed with water, dried over MgSO_4_, filtred and evaporated. The residue was purified by chromatography on silica gel plates with CH_2_Cl_2_-(CH_3_)_2_CN (95:5) as eluent and the (X_4_Me_2_Q_2_) isolated as yellow powder (0.34 g, 79 %). The final product is characterized, in first step by Thin Layer Chromatography: TLC (SiO_2_, eluent CH_2_Cl_2_-(CH_3_)_2_CO, 90/10, v/v) R_F_ = 0.85; in the second step by MALDI-TOF Mass Spectroscopy (Fig. 1): m/z (relative intensity): 594 (M+2H^+^); 615 (M+Na^+^); 631 (M+K^+^) and finally by ^1^H NMR spectroscopy (Fig. 2) (300 Hz, CDCl_3_): *δ* (ppm) 1.34 [s, 18H, C(CH_3_)_3_]; 3.10 [s, 6H, OCH_3_]; 3.32 [br s, 4H, ArCH_2_Ar]; 3.78 [br s, 4H, ArCH_2_Ar]; 6.32 [s, 4H, QuH]; 7.22 [s, 4H, ArH]. The schematic pathway of synthesis of the X_4_Me_2_Q_2_ has been reported in our previous work [25].

### Electrochemical investigations

A typical electrochemical impedance experimental set-up consists of an electrochemical cell and a Tacussel PGP 201 potentiostat/galvanostat. A three-electrode configuration for an electrochemical cell is the most common for typical electrochemical applications. The thin film was electrodeposited on the working electrode. The second functional electrode is the counter electrode, which serves as a source sink for electrons so that the current can be passed from the external circuit through the cell. The third is the reference electrode, in which the potential is constant enough and can be taken as the reference standard. The electrode is used to determine the potential of the working electrode precisely. Since the absolute potential of a single electrode cannot be measured, all potential measurements in electrochemical systems are performed with respect to a reference electrode. A reference electrode, therefore, should be reversible, and its potential should remain constant during the course of the measurement.

Indium–tin oxide glass plates (ITO thickness 100 nm, sheet resistance 20 Ωcm) were purchased from Merck Display Technologies and are cut into 2 × 1 cm square slides and was served as work electrode. Prior to thin layer electrodeposition, the ITO substrates are successively cleaned in water then in acetone and finally dried at 60 °C.

Tetrabutylammonium perchlorate TBAP (Fluka) was purified by recrystallization from ethanol and was used as supporting electrolyte. The acetonitrile 99 % was purchased from Acros Organics and was used as received.

The electrochemical set-up consisted of a Tacussel (PGP 201) potentiostat.

The auxiliary electrode is the same nature as that of work. As reference electrode, we use the saturated calomel (SCE).

All the experiments were carried out at laboratory temperature. Solutions containing TBAP (0.1 M) as supporting electrolyte were protected from atmosphere with argon prior to each cyclic voltammetry measurement and the gas flow maintained during the CV experiments.

## Results and discussion

### Investigation in acetonitrile solvent

#### Electrochemical behavior of the ITO electrode

**Fig. 3:**
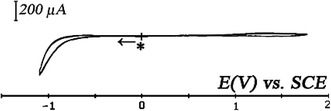
Cyclic voltammetry at ITO coated in electrode in electrolyte support {CH_3_CN + 0.1 M TBAP} solution, sweep rate: *v* = 0.025 Vs^−1^. *Asterisk* denotes the start and the final potential

Cyclic voltammogram of the ITO glasses in contact with a solution of {CH_3_CN + 0.1 M TBAP}, is recorded to find out the electroactivity domain of the ITO layer. By varying the potential scan (Fig. 3), we see that the electroactivity domain ranges from −1.30 V versus SCE to 1.80 V versus ECS (sweep rate *v* = 0.025 Vs^−1^). No additional peaks can be observed, indicating that no redox reaction has occurred.

#### Cyclic voltammetry of calix[4]arenediquinone (X_4_Me_2_Q_2_) in acetonitrile

**Fig. 4:**
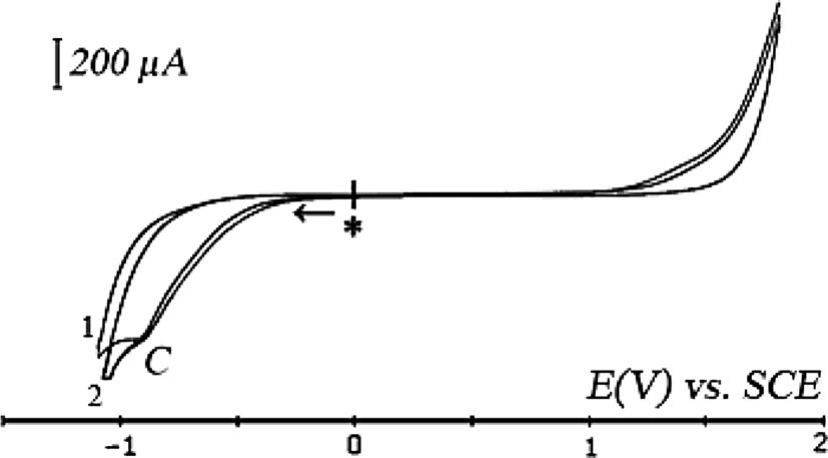
Cyclic voltammogram at ITO coated electrode of 8 × 10^−3 ^M of X_4_Me_2_Q_2_ in {CH_3_CN + 0.1 M TBAP}. Sweep rate: *v* = 0.025 Vs^−1^. *Asterisk* denotes the start and the final potential

The electrochemical activity of X_4_Me_2_Q_2_ (7.88 × 10^−3^ M), was investigated by cyclic voltammetry at ITO working electrode in {CH_3_CN + 0.1 M TBAP}. Figure 4 shows two successive cyclic voltammograms of X_4_Me_2_Q_2_ at 0.025 Vs^−1^. The CV shows one cathodic peak at potential of E_p_(C) = −0.93 V versus SCE in the reduction zone. By comparing with the voltammogram unregistered for ITO electrode in the supporting electrolyte, this phenomenon could be attributed to reduction of calix[4]arenediquinone [25, 32].

#### Preparative electrolysis and coulometry

Macroscale electrolysis of X_4_Me_2_Q_2_ (46.5 mg; 0.078 mmol; electrolysis duration 23 min) was accomplished at a potential slightly more negative than the cathodic wave (about −1.00 V vs SCE). Homogenization of the electrolytic solution was ensured by a mechanical stirring. After 23 min (t = 1380 s), we stopped the electrolysis with a constant current equal to *i* = *5.75* × *10* ^−*3*^ *A.*

A brown self-assembled adlayers was detected on the surface of the ITO electrode. When the current is held strictly constant during an electrolysis the quantity *Q* of electricity passed is simply *Q* = *i* × *t* Coulombs, where *i* is the current in Amperes and *t* the time in second. The quantity of coulomb into play during the electrodeposition film is given by the equation *dQ* = *i* × *dt.* The integration of the area of a rectangle allows us access to the amount of coulomb consumption: *Q* = *7.9* Coulombs.

We note that the glass electrodes are not degrading over the course of the experiments. The conductivity of the ITO electrode was controlled and measured before and after electrolysis in the electrochemical using voltmeter.

#### Cyclic voltammetry of the recovered electrode

**Fig. 5:**
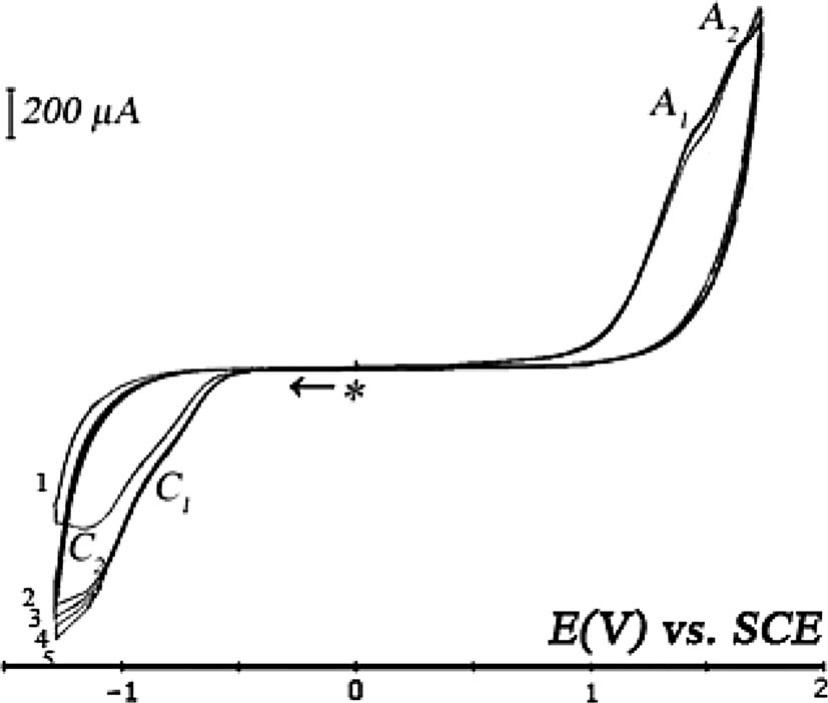
Cyclic voltammogram at ITO coated electrode recovered with deposit in electrolyte support {CH_3_CN + 0.1 M TBAP} solution. Sweep rate: *v* = 0.025 Vs^−1^. *Asterisk* denotes the start and the final potential

Figure 5 shows the cyclic voltammgram of the calix[4]arene self organized on ITO electrode in {CH_3_CN + 0.1 M TBAP} electrolyte support solution. Two irreversible cathodic peaks appears at potentials E_p_(C_1_) = −0.79 versus SCE and E_p_(C_2_) = −1.14 V versus SCE. They are probably related to the reduction of calix[4]arenediquinone with a slight shift to more positive potentials compared to their positions on the voltammogram recorded for a platinum disk electrode [25] or else for a glassy carbon electrode [33].

Two irreversible anodic oxidation peaks appear at potentials respectively E_p_ (A_1_) = 1.44 versus SCE and E_p_ (A_2_) = 1.60 V versus SCE with high intensity. In fact, these two waves could be assigned to the oxidation of *tert*-butyl anisole [34] with a shift of the potential which is probably to the conductive layer of ITO. In comparison with the voltammogram recorded with an ITO electrode in contact with the supporting electrolyte solution (Fig. 3), we can conclude that the conductive surface of the electrode is sufficiently recovered.

#### UV–Vis Spectra properties of the calix[4]arene auto-assembled on ITO electrode

**Fig. 6:**
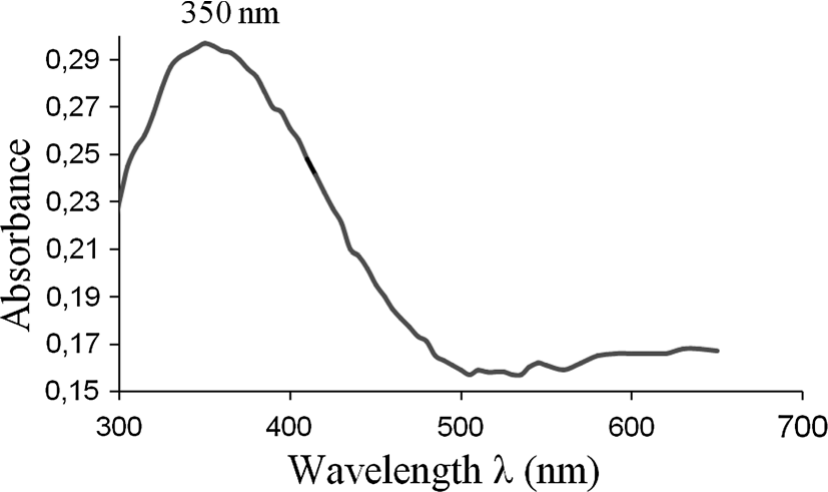
UV–Vis spectra of the self-organized brown film on ITO coated electrode in the acceptor regions

UV–Vis spectra of a calix[4]arene derivative auto-assembled electrochemically in acetonitrile solvent on thin film sample has been performed. The spectrum exhibits one significant absorption band at 350 nm as shown in Fig. 6. The absorption band arises on the first absorption band of the acceptor in the calix[4]arene, it may be attributed to calix[4]arenediquinone or calix[4]arenedihydroquinone electrogenerated during the partial reduction of the substrate. The spectra don’t show any absorption in the region of the charge-transfer band.

#### Chromatography analysis

The electrolytic solution was analysed by thin layer chromatography on a silica plate before and after electrolysis (eluent CH_2_Cl_2_/(CH_3_)_2_CO, 95/5, v/v). The revelation is made under a UV lamp (λ = 254 nm). The chromatogram realized before electrolysis to identify a single substance which is our starting substrate (R_F_ = 0.78). Nevertheless, that achieved after electrolysis, highlights the presence of three substances that are referring to work done in our laboratory [26].

##### Substance 1

(R_F_ = 0.78) corresponding to the starting material: X_4_Me_2_Q_2_.

##### Substance 2

(R_F_ = 0.58) there is an intermediate compound, the charge-transfer complex that would be formed during the partial reduction of the starting material: X_4_Me_2_Q(H_2_Q) [26].

##### Substance 3

(R_F_ = 0.34) is the oxidized form of the starting material: X_4_Me_2_(H_2_Q)_2_ [26].

This analysis enlighten that the charge-transfer complex: the calix[4]arenequinhydrone X_4_Me_2_Q(H_2_Q) is formed in solution and not electrochemically deposited on ITO electrode under the recommended conditions.

#### Scanning electron microscopy

**Fig. 7:**
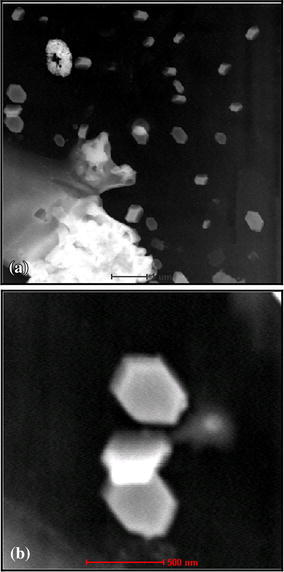
SEM images of the modified electrode. **a** 1 μm enlargement; **b** 500 nm enlargement

The presence of the calix[4]arene film on ITO electrode surface can be further confirmed by SEM photograph. The images are presented within Fig. 7. The functionalized calix[4]arene displayed an hexagonal structure. Although there were small amount of product auto-assembled, a clear layer was obvious. This indicate that calix[4]arenes molecules has been assembled on the ITO electrode surface, corresponding to the result from cyclic voltammetry.

### Investigation in dichloromethane solvent

#### Electrochemical behavior of the ITO electrode

Cyclic voltammogram of the ITO glasses in contact with a solution of {CH_2_Cl_2_ + 0.1 M TBAP} is recorded No additional peaks can be observed, indicating that no redox reaction has occurred.

#### Cyclic voltammetry of calix[4]arenediquinone (X_4_Me_2_Q_2_)

**Fig. 8:**
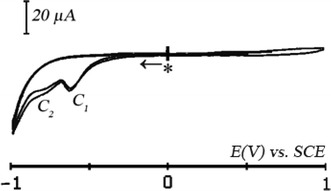
Cyclic voltammogram at ITO coated electrode of 7.88 × 10^−3^ M of X_4_Me_2_Q_2_ in {CH_2_Cl_2_ + 0.1 M TBAP}. Sweep rate: *v* = 0.05 Vs^−1^. *Asterisk* denotes the start and the final potential

The cyclic voltammetry investigation at ITO electrode were carried out in electrolytic solution containing {CH_2_Cl_2_ + 0.1 M TBAP} and X_4_Me_2_Q_2_ (7.88 × 10^−3 ^M). At a scan rate of 0.050 Vs^−1^, Fig. 8 exhibits one well defined reduction peak (C_1_) at a potential of E_p_(C_1_) = −0.63 V versus SCE that is followed by a small wave (C_2_) at a potential of E_p_(C_2_) = −0.80 V versus SCE. These two peaks are probably related to the double reduction of the substrate. No peak appears in the oxidation zone.

#### Preparative electrolysis

Electrochemical technologies attract constantly rising interest because it is the way for controlled modifications of the surface even with atomic precision. In recent years, more attention has been paid to electrochemical deposition technique for manufacturing thin films and devices of carbon based materials due to its simplicity, its low capital equipment cost, and its ability to be scaled up for large production [35–37]. In previous work, the calix[4]arene molecule has been dispersed in a conjugated polymer and deposited by spin coating or evaporation process onto the ITO glass [39, 40]. In our study, we use the electrochemical method for the calix[4]arene deposition.

The electrolysis of the substrate was achieved at potentials slightly more negative than their corresponding to the first cathodic peak (−0.65 V vs SCE; 1.6 × 10^−2^ mol L^−1^; duration: 6 h).

A blue self-assembled adlayers was detected on the surface of the ITO electrode. At first sight, the colour of the self-assembled layer alluded to the charge-transfer complex detected in recent studies [27] in dichloromethane solvent.

#### Cyclic voltammetry of the recovered ITO coated electrode

**Fig. 9:**
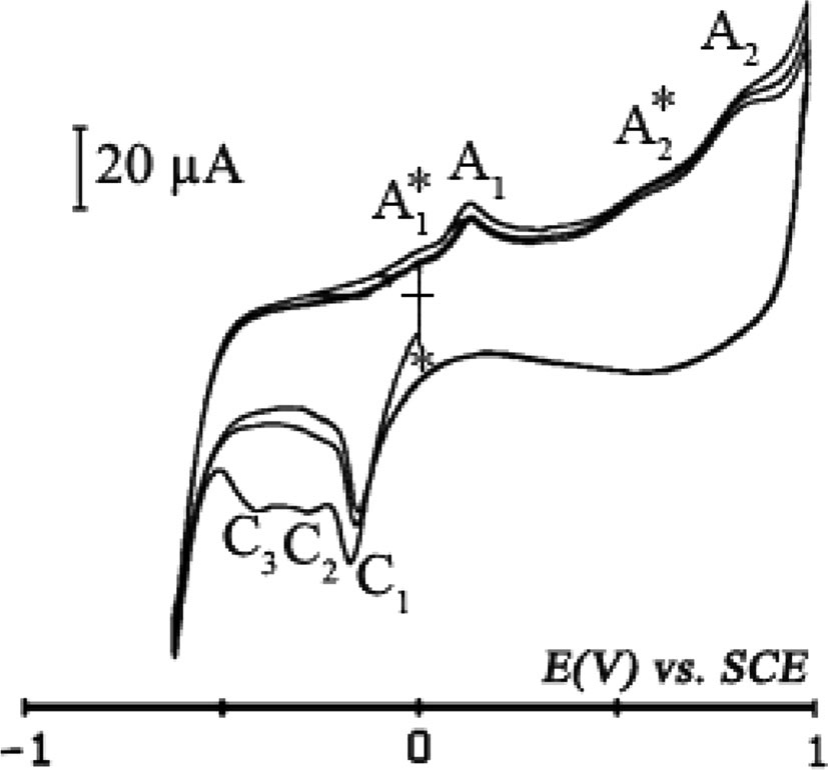
Cyclic voltammogram at ITO coated electrode recovered with deposit in electrolyte support {CH_2_Cl_2_ + 0.1 M TBAP} solution. Sweep rate: *v* = 0.025 Vs^−1^. *Asterisk* denotes the start and the final potential

Coated electrode was rinsed with dichloromethane to remove the electrolysis solution excess. A cyclic voltammetry of the ITO electrode coated with the bluish deposit, is accomplished by contacting a supporting electrolyte solution {CH_2_Cl_2_ + 0.1 M TBAP}. Figure 9 exhibits one well-defined cathodic irreversible peak (C_1_) followed by two waves (C_2_) and (C_3_). These peaks may correspond to the reduction of the calix[4]arenediquinone [25]. In the oxidation compartment, two peaks appear (A_1_) and (A_2_), both are preceded by a wave of low intensity (A_1_^*^) and (A_2_^*^). Potential values on the first cycle are summarized in Table 1. The peak (A_2_) appears with a well-defined intensity indicates the presence of calix[4]arenedihydroquinone. This result is similar to that presented in the literature [25]. However, this electrochemical behavior confirms the self-assembled calix[4]arenediquinone/calix[4]arenedihydroquinone assembly on the ITO coated electrode.

**Table 1:** Cyclic voltammetry patterns of ITO coated electrode recovered with deposit in electrolyte support {CH_2_Cl_2_ + 0.1 M TBAP} solution

Pics C_i_, A_i_	C_1_	C_2_	C_3_	A_1_^*^	A_1_	A_2_^*^	A_2_
E(V) versus SCE	−0.16	−0.28	−0.42	0.00	0.13	0.56	0.87

#### UV–Vis Spectra properties of the calix[4]arene auto-assembed on ITO coated electrode

**Fig. 10:**
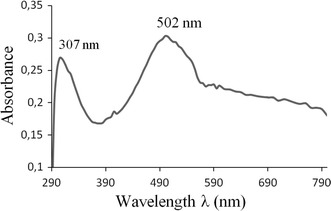
UV–Vis spectra of the self-organized blue film on ITO coated electrode in the acceptor and charge-transfer regions

Figure 10 shows the absorption spectrum of the blue film electrodeposited on the ITO electrode after rinsing with dichloromethane. At least two contributions peaked, in the UV range at 307 nm and in visible range at 502 nm, are needed to reproduce the absorption band which are separated by around 200 nm. We attribute the two measured absorption bands, respectively, to an acceptor band and to a complex charge-transfer band. The presence of complex charge-transfer band suggests the presence of the calix[4]arenequinone/hydroquinone or calix[4]arenequinhydrone X_4_Me_2_Q(H_2_Q) [8, 38]. Previous work [27] showed that whatever the solvent, the calix[4]arenequinhydrone absorbs in the visible (Acetonitrile: λ = 580 nm, dichloromethane: λ = 632 nm) while the reduced form X_4_Me_2_Q_2_ and the oxidized form X_4_Me_2_(H_2_Q)_2_ absorb in the ultra-violet range. This finding agree with a previous results, even if the calix[4]arenequinhydrone is chemical synthesized.

Film auto-assembled from the CH_2_Cl_2_ (dielectric constant ε_r_ = 9.1; dipolar moment μ = 1.60 D) electrolytic solution shows a strong blue coloration associated with the existence of a charge-transfer complex. By comparison, film auto-assembled from the CH_3_CN (dielectric constant ε_r_ = 37; dipolar moment μ = 3.92 D) electrolytic solution reveals a brown coloration and the absorption spectrum don’t prove any presence of the charge-transfer complex. These observations let us conclude that the solvent polarity present a great effect on the formation of the charge-transfer complex. In fact, it is well known that a transition between two states with different charge distributions depends on the polarity of the solvent in which is determined. The effect of increasing the solvent polarity increases the stability of the most polar state [41].

## Conclusion

The partial electrochemical reduction of di(methoxy-*p*-*tert*-butyl)calix[4]arenediquinone leads to the formation of the oxidative form calix[4]arenedihydroquinone. The presence of both entities in the solution generates the charge-transfer complex the calix[4]arenequinhydrone at the ITO coated electrode surface through a process of the donor/acceptor between calix[4]arenequinone/calix[4]arenedihydroquinone assembly. Besides, the voltammetric patterns applied for the auto-assembled film on ITO coated electrode confirm the existence of X_4_Me_2_(H_2_Q)_2_/X_4_Me_2_Q_2_ assembly. Based on the UV–Vis spectra investigation, we can tell from the spectrum recorded that the first absorption band appeared in the UV range is related to the presence of X_4_Me_2_Q_2_ or/and X_4_Me_2_(H_2_Q)_2_ and the band appeared in visible range is related to the charge-transfer complex calix[4]arenequinhydrone X_4_Me_2_Q(H_2_Q). The film auto-assembled from the CH_2_Cl_2_ electrolytic solution shows a strong blue coloration associated with the existence of a charge-transfer complex. However, comparing with brown film auto-assembled from the CH_3_CN electrolytic solution, the absorption spectrum doesn’t prove any presence of the charge-transfer complex. This funding let us conclude that the formation of the charge-transfer complex depend of the solvent polarity. Thus, the imposed potential electrolysis in dichloromethane is an interesting way to synthesize electrochemically charge-transfer complex, the calix[4]arenequinhydrone. To build electrochemically modified electrodes with calixarenes, it was appropriate to apply a cathodic reduction to promote the self-assembly on ITO electrode conductive layer.
